# Seeing the Forest for the Trees, and the Ground Below My Beak: Global and Local Processing in the Pigeon’s Visual System

**DOI:** 10.3389/fpsyg.2022.888528

**Published:** 2022-06-09

**Authors:** William Clark, Michael Colombo

**Affiliations:** Department of Psychology, University of Otago, Dunedin, New Zealand

**Keywords:** fovea, tectofugal, thalamofugal, neural coding, wulst, image perception, hippocampus, dorsal ventricular ridge

## Abstract

Non-human animals tend to solve behavioral tasks using local information. Pigeons are particularly biased toward using the local features of stimuli to guide behavior in small-scale environments. When behavioral tasks are performed in large-scale environments, pigeons are much better global processors of information. The local and global strategies are mediated by two different fovea in the pigeon retina that are associated with the tectofugal and thalamofugal pathways. We discuss the neural mechanisms of pigeons’ bias for local information within the tectofugal pathway, which terminates at an intermediate stage of extracting shape complexity. We also review the evidence suggesting that the thalamofugal pathway participates in global processing in pigeons and is primarily engaged in constructing a spatial representation of the environment in conjunction with the hippocampus.

## Introduction

Behavioral tasks can be solved in either absolute or relative manners. Take, for example, an elegant series of studies conducted by D’Amato and his colleagues (D’Amato and Salmon, 1984; D’Amato, 1988; D’Amato and Colombo, 1988). They trained monkeys to discriminate between two tunes, easily recognizable as such to any human. The question is whether the monkeys integrated the entire tune, what we would call a *relative-* or *global-feature* solution, or whether they based the discrimination of the two tunes on local features, what we would call an *absolute-* or *local-feature* solution. For ease of exposition, hereafter we will simply refer to the two solution methods as either *global* or *local*.

One way to distinguish whether the monkeys used a *global* or *local* solution in the tune discrimination is to perform an octave transformation, that is, increase the frequencies of all the notes in the tunes by an octave. To a *global* processor, although the tunes would now carry a higher overall frequency, you would still be able to tell the two tunes apart. To a *local* processor, however, it would now be difficult to tell the two tunes apart. The reason is because a *local* processor would have based the discrimination between the two tunes on local features, say the frequency of the last note in both tunes, and changing the tunes by an octave would have disrupted that frequency-dependent “local” solution. Indeed, while we humans would have little difficulty in telling the octave-transformed tunes apart, monkeys were no longer able to discriminate between the two tunes.

The reliance on local features within a stimulus, as described in the tune discrimination situation, can also lead to local/global failures on a more conceptual level. Take, for example, the matching concept, and whether animals have concepts of “same” and “different.” Many years ago, Weinstein (1941) tested rhesus monkeys and children on a matching task often used to determine whether animals have a matching concept. The essence of a matching task is to train subjects to play a same/different task with two stimuli, and once they learn to play the task with the two stimuli, test them with two novel stimuli. If the subjects continue to play the game with the novel stimuli we call them *global* processors, and in possession of an abstract concept of “same” and “different,” whereas if they struggle with the novel stimuli they are more likely *local* processors, having learned to play the task by a set of stimulus–response rules specific to the original training stimuli.

Weinstein (1941) found that the ability of monkeys to transfer to novel situations was far more restricted than the children. For example, both monkeys and children were originally trained with three-dimensional objects, and both transferred to novel three-dimensional objects, although the children were slightly better than the monkeys. The gap between the children and monkeys increased substantially when the shapes were changed from three-dimensional objects to two-dimensional objects, and increased even more when the response was changed from pushing aside a stimulus to lifting a lid to expose a stimulus, a response that effectively has little bearing to the actual solution of the task. Yet the monkeys struggled with such a small change. The reason, of course, is that the monkeys failed to pick up on the global aspects of the task and focused instead on many local features of the stimuli, some that were even irrelevant to the solution of the task (e.g., the manner of responding).

The comparative cognition literature is replete with examples of animals relying on local features of stimuli, and by extension, local solutions to problems at a more conceptual level. We do not deny that there are species differences in the ability to engage local or global features of stimuli (Clayton and Krebs, 1994a,b), and we do not deny that the ability to extract local or global features can be highly task-dependent and conditional on training (Fremouw et al., 1998; Rosa Salva et al., 2013), but when pitted against each other, animals often opt to process information at a local level. And although the range of situations to which monkeys will transfer a behavior is limited, pigeons fare even worse. It is not that pigeons cannot be trained to be global processors. Indeed, we have shown that pigeons can perform the same tasks as monkeys do, and to the same levels (Colombo et al., 2003; Scarf et al., 2011, 2016). The difference is that one must take extra measures with pigeons to design the experiment in such a way that minimizes the reliance on local cues (Colombo and Scarf, 2020). It is as if animals differ not in their ability to perform a task, but in the degree to which they are *global* and *local* processors, with pigeons representing a species that is firmly in the *local* processor end of the *global/local* spectrum. The question is: *Is there a neural basis for this reliance on local cues?* We believe that there is.

## An Overview of Pigeon Vision

**Figure 1:**
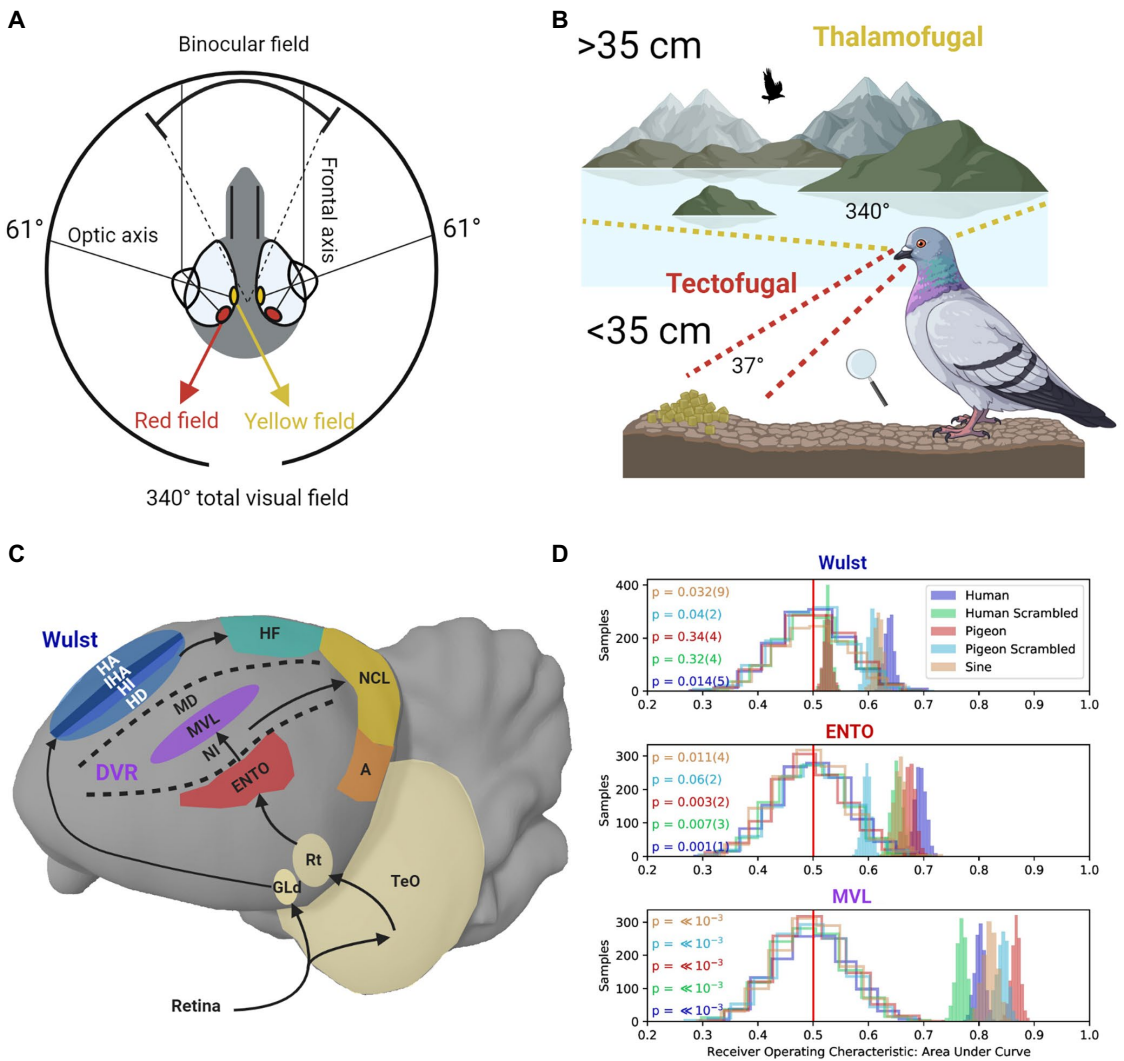
**(A)** The pigeon eye has two fovea regions that are located close to the optic axes. The yellow field is mainly used to view distant objects in the monocular lateral visual field. The red field is mainly used to view nearby objects. The frontal axes converge during viewing of nearby objects, producing a field of binocular overlap. **(B)** Frontal viewing of nearby objects using the red field is mainly performed using the tectofugal pathway (red lines). Lateral viewing of distant objects using the yellow field is mainly performed using the thalamofugal pathway (yellow lines). **(C)** Sagittal depiction of visual information flow in the tectofugal pathway (Red, Purple) layers of the DVR to NCL (Yellow), and thalamofugal pathway layers (Blue) to HF (Green). GLd, dorsolateral geniculate nucleus; HA, hyperpallium apicale; IHA, interstitial nucleus of the hyperpallium apiciale; HI, hyperpallium intercalatum; HD, hyperstriatum dorsale; HF, hippocampal formation; TeO, optic tectum; Rt, nucleus rotundas; ENTO, entopallium; NI, intermediate nidopallium; MVL mesopallium ventrolaterale; MD, mesopallium dorsale; NCL, nidopallium caudolaterale; A, arcopallium; DVR, dorsal ventricular ridge. **(D)** Single-unit recordings during frontal viewing of images determined that MVL of the tectofugal pathway discriminates between different images with high capacity, in contrast with ENTO and the Wust (Clark et al., 2022). The unfilled distributions show the performance of a linear discriminant analysis trained on randomly labeled firing rate data, which contain “no information” for the permutation significance test. The shaded distributions show the performance of correctly labeled data. *p* values (and their error) are shown to the left for each group of stimuli viewed by the pigeons (color coded on the left). The value of *p* for each stimulus group is derived from how far away from the “no information” distribution of samples that the correctly labeled performance falls.

Like many granivorous birds with laterally placed eyes, pigeons need to detect grain against a textured surface at close range while also searching for predators, monitoring conspecifics behavior, and scanning the visual field during flight (McFadden et al., 2001; Fernández-Juricic et al., 2004; Delius and Delius, 2019). To facilitate these contradictory demands on the visual system, the pigeon eye represents a 37° field of binocular overlap near the frontal eye-beak axis, and a monocular visual field covering 340° (Hayes et al., 1987; see Figure 1A). Two different fovea in the pigeon retina with enhanced ganglion cell density and differently colored oil droplets enhance spatial resolution (Nalbach et al., 1990; Letelier et al., 2004). The red field fovea mediates high-resolution vision in the binocular frontal visual field (Hayes et al., 1987). When pigeons view a nearby object on the ground their eyes converge, and the red field is used to guide accurate pecks toward a target (Goodale, 1983; see Figure 1B). In contrast, the yellow field fovea is responsible for high-resolution vision in the monocular lateral visual field (Hahmann and Güntürkün, 1993; see Figure 1B).

The red field and yellow field are also associated with the two main visual pathways in the pigeon brain. The tectofugal visual pathway in laterally eyed birds (similar to the mammalian colliculo-pulvinar-cortical pathway) is primarily involved in pattern vision associated with information in the red field (Hodos et al., 1988; Wang et al., 1993; Hellmann and Güntürkün, 1999). The thalamofugal visual pathway (similar to the mammalian geniculo-striate pathway) primarily mediates visuo-spatial localization (Budzynski et al., 2002; Watanabe et al., 2011) and pattern vision associated with yellow field information (Güntürkün and Hahmann, 1999; Budzynski and Bingman, 2004). Given that most behavioral testing of pigeons takes place in an operant chamber, there is a strong bias in such an environment toward using the red field fovea, and hence the tectofugal system.

## Functional Organization of the Tectofugal Visual Pathway

In primates, there is a multi-stage progression of representations along the ventral stream beyond the primary visual cortex (V1; DiCarlo et al., 2012). At the level of V1 and neighbouring regions, receptive fields are small and retinotopically organized (Schiller et al., 1976; Engel et al., 1997). As one moves progressively further away from V1, receptive fields become larger, and global representations of object defining features that are tolerant to non-linear changes (such as viewpoint, illumination, and translation) emerge at higher ventral stream stages like inferior temporal (IT) cortex (Gross, 1992; Gochin et al., 1994; Freiwald and Tsao, 2010; Bao et al., 2020).

Much like the primate ventral stream, the avian tectofugal pathway is also organized in a similar series of hierarchical processing stages. Visual information from the pigeon retina is sent to the contralateral optic tectum, which consists of five different types of tectal ganglion cells that extract form, color, and motion information (Hellmann et al., 2004). Tectal ganglion cells in the deep layers of the optic tectum project in parallel to the nucleus rotundus of the thalamus (Hellmann and Güntürkün, 2001). Nucleus rotundus forwards information to the entopallium (ENTO), which is involved in shape identification and motion perception in the telencephalon (Nguyen et al., 2004; Krützfeldt and Wild, 2005).

There are similarities with the primate visual cortex with respect to an increase of receptive field sizes and the complexity of information that is coded at different levels of the tectofugal pathway. Receptive field sizes and the complexity of response properties increases between the superficial and deep layers of the optic tectum (Jassik-Gerschenfeld and Guichard, 1972; Frost and DiFranco, 1976). Receptive field sizes at the levels of nucleus rotundas and ENTO are also large, and ENTO displays subdivisions for the selective processing of form, color, and motion information (Wang et al., 1993; Nguyen et al., 2004).

With respect to processing global shape information in the tectofugal pathway, during frontal viewing of object stimuli in an operant chamber, ENTO neurons’ population responses do not distinguish well between the features of different stimuli (Azizi et al., 2019; Clark et al., 2022; see Figure 1D). Information from ENTO is then sent to MVL in the mesopallial layers (Stacho et al., 2020; see Figure 1C). Azizi et al. (2019) demonstrated that the population response of MVL distinguishes between the features of animate and inanimate objects with high accuracy. In addition, the MVL population response differed from a model of simple V1-like edge detectors with respect to the image features they used to achieve categorization of the objects. These findings suggest that visual information is recoded between ENTO and MVL in a hierarchical manner, and some degree of non-linear operations represent features more abstract than oriented edges.

Clark et al. (2022) also found that the population response of MVL distinguished between the features of different images with greater capacity than at the level of ENTO (Figure 1D). However, they also found that many of the MVL neurons displayed strong responses to scrambled images, analogous with those observed in primate V1 (Rainer et al., 2002) and rodent V1 (Vinken et al., 2016). MVL showing strong responses to scrambled images is different from the mammalian system, where visual areas beyond V1 do not show strong responses to scrambled images. These observations suggest that local edges and some more abstract (global) features of stimuli are processed by an MVL population. The complexity of representation emerging at the level of MVL may be most similar to that found in rodent extrastriate cortex. For example, while there are increasingly abstract shape computations along rodent extrastriate cortex (Marshel et al., 2011; Vermaercke et al., 2014; Matteucci et al., 2019), like pigeon MVL, the representation does not reach a stage of complexity comparable to that observed in primate IT cortex (Vinken et al., 2016; Vinken and Op de Beeck, 2021).

What might these observations mean with respect to pigeons’ visual behavior? When pigeons scrutinize nearby images in an operant chamber using the red field, they engage the tectofugal system, where higher stages (such as MVL) are less prone to global processing than regions in the primate ventral stream (Cavoto and Cook, 2001; Cook and Hagmann, 2012; Cook et al., 2015; Murphy et al., 2015). The primary ecological function of the red field is mainly for the detection of grain against a textured surface (Figure 1B) to guide bill strikes (Goodale, 1983; Delius and Delius, 2019). These specializations may favor the additive use of sharp edges and fine (local) details, whereas global stimulus processing can be accomplished without needing a stage of complexity comparable to what is seen in primate IT cortex.

## What Happens When a Pigeon Looks at a Distant Object Laterally in the Yellow Field? The Role of the Thalamofugal Pathway and Hippocampus

When pigeons view distant objects, visual information from the yellow field (Figure 1A) is represented by the dorsolateral geniculate nucleus (GLd), which projects to the visual Wulst in the telencephalon (Remy and Güntürkün, 1991; see Figure 1C). In contrast, the red field is only represented to a very limited extent by GLd (Remy and Güntürkün, 1991), reinforcing the notion that the yellow field, and its representation at the level of GLd, is primarily engaged in high-resolution vision at a distance. Highlighting the dual nature of the thalamofugal and tectofugal pathways, lesions applied to GLd selectively reduce the visual acuity of the yellow field, but not the red field (Güntürkün and Hahmann, 1999).

Ortega et al. (2008) showed that pigeons trained to discriminate between two shapes are severely impaired in their performance when the stimuli were moved from the red field into the yellow field. These findings support the view that there is almost a complete dissociation of stimulus information when switching viewing from the tectofugal to the thalamofugal pathway. The division between the visual pathways likely facilitates manipulation of nearby food while also monitoring laterally for potential predators. There was, however, some intraocular transfer of stimulus information when switching from the thalamofugal to the tectofugal system. These findings likely reflect the ecological requirement for distant objects (e.g., grain among other plant material) first viewed in the yellow field to be transferred into the representation of the red field proximally.

The visual Wulst is likely homologous with V1 and displays some similarities in laterally eyed birds. These include orientated edge detectors (Ng et al., 2010), flexible reward and stimulus association coding (Anderson et al., 2020), and retinotopically organized maps of visual space (Revzin, 1969; Gusel’nikov et al., 1977; Bischoff et al., 2016). There is also evidence that the Wulst is involved in representing global spatial information in comparison with the tectofugal pathway, as opposed to local beacons. Wulst lesions in pigeons disrupt the integration of polarized light information about the sun’s azimuth, which is important for pigeons to determine their position in space and identify a goal direction (Budzynski et al., 2002). Budzynski and Bingman (2004) also demonstrated that lesions to the pigeon Wulst impair performance in discriminations of oriented gratings in an open field area, which surely would recruit the yellow field, but not in an operant chamber when viewed using the red field.

Layer hyperstriatum dorsale of Wulst forms a major reciprocal connection with the dorsolateral subdivision of the hippocampal formation (Atoji et al., 2018; see Figure 1C). Like the mammalian hippocampus, the avian hippocampus is primarily involved in the integration of sensory features into a spatial representation of the environment (Colombo and Broadbent, 2000; Mouritsen et al., 2016; Johnston et al., 2020; Ben-Yisahay et al., 2021; Payne et al., 2021). The global processing of spatial landmarks based on yellow field information from the thalamofugal pathway is mainly dependent on the hippocampus. Interestingly, the effects hippocampal lesions have on position discrimination (Broadbent and Colombo, 2000) and radial arm maze analogue tasks (Colombo et al., 1997) are generally greater when pigeons perform these tasks in large-scale environments compared to small-scale environments such as inside an operant chamber, a condition referred to as the Big-Box-Little-Box effect (Colombo and Broadbent, 2000; Johnston et al., 2020).

Clearly the thalamofugal pathway is important for distance vision, but at the level of Wulst and the hippocampus, spatial localization appears to take on a prominent role over detailed shape analysis, consistent with the findings that detailed vision as assayed by operant chamber tasks is generally not affected by a lesion of these structures. Further consistent with this notion is the absence of foveal magnification to enhance the resolution of the system, as seen in the zebra finch Wulst (Michael et al., 2015; Bischoff et al., 2016). The absence of foveal magnification further suggests that spatial localization of objects using global scene information may be the primary role of the Wulst in laterally eyed birds. Consistent with a primary role in spatial localization, zebra finches with Wulst (Watanabe et al., 2011) and hippocampal (Watanabe et al., 2008) lesions display deficits in spatial discriminations, but not in pattern discriminations.

A future avenue of research will be to examine how hierarchal processing of visual stimuli to form global representations of the environment is mediated at different stages of the thalamofugal pathway leading up to the hippocampus. Intriguingly, Damphousse et al. (2022) recently showed that lesions to the area parahippocampalis (including the dorsolateral hippocampal subdivision) in quail impairs object recognition as well as spatial processing, whereas medial hippocampus lesions only impair spatial behavior. We speculate that the impairments observed in both object recognition and spatial foraging tasks after lesions to area parahippocampalis might be related to its association with the visual Wulst. Specifically, the Wulst may process shape and spatial information viewed in the yellow field, and relay both types of information to the hippocampus *via* the hyperstriatum dorsale. Another important region that contributes to global processing is the nidopallium frontolaterale, which represents information associated with the features of different training environments (Gao et al., 2019) and sends integrated visual information from the tectofugal and thalamofugal pathways to the dorsolateral hippocampal subdivision directly, and indirectly *via* hyperstriatum dorsale (Atoji et al., 2018).

## Summary

Pigeon’s red field and yellow field are adapted for local and global viewing modes, and these specializations are reflected by the physiology of the tectofugal and thalamofugal pathways. Most behavioral experiments using pigeons take place in an operant chamber, which bias them toward a solution mediated by the tectofugal pathway and hence local features. As a result, pigeons performing tasks in operant chambers tend toward being *local* processor of information. Although the thalamofugal pathway mediates more global processing, we suspect that the type of global processing undertaken by this system is quite different from that seen in primates. In primates, the thalamofugal system (specifically the ventral stream prior to the parahippocampal cortex) is primarily related to processing global stimulus features independent of the context in which it occurs (Hung et al., 2005; Rust and DiCarlo, 2010; Aminoff et al., 2013), whereas in birds we suspect that the “global” processing relates more to how that visual information is embedded in large-scale environments, a type of allocentric representation essential to a bird’s navigation.

## Author Contributions

WC and MC researched the concept and wrote the manuscript. All authors contributed to the article and approved the submitted version.

## Funding

This work was supported by a Royal Society of New Zealand Marsden Fund grant 19-UOO-162 to MC.

## Conflict of Interest

The authors declare that the research was conducted in the absence of any commercial or financial relationships that could be construed as a potential conflict of interest.

## Publisher’s Note

All claims expressed in this article are solely those of the authors and do not necessarily represent those of their affiliated organizations, or those of the publisher, the editors and the reviewers. Any product that may be evaluated in this article, or claim that may be made by its manufacturer, is not guaranteed or endorsed by the publisher.
